# Hepatitis B immunity among undergraduate nursing students: A brief report

**DOI:** 10.1111/phn.13148

**Published:** 2022-11-09

**Authors:** Robin M. Dawson, Courtney Catledge, Ann Scott, Leigh Blackmon Pate, Gail Vereen

**Affiliations:** ^1^ University of South Carolina College of Nursing Columbia South Carolina; ^2^ USCL BSN Collaborative Program University of South Carolina – Lancaster Lancaster South Carolina

**Keywords:** hepatitis, nursing education, public health nursing education, vaccination

## Abstract

**Background:**

Health sciences students who report low/equivocal hepatitis B titers may be required to repeat the immunization series, even though the result may not indicate non‐immunity.

**Method:**

To describe hepatitis B immunity patterns, this retrospective, descriptive study utilized de‐identified vaccination records and anti‐HBs titers of three cohorts of sophomore nursing students entering clinical rotations in 2018–2019.

**Results:**

Only 33% of students had initial anti‐HBs quantitative serum titer ≥10 mIU/ml, demonstrating immunity. After students with low/equivocal titers (n = 191, 64%) were re‐immunized per institution protocol, only 2% (n = 7) were identified as non‐responders. Cumulative costs incurred by students for revaccination and repeat titer exceeded $20,000, with a process time of up to 8 months.

**Conclusion:**

While rates of exposure to hepatitis B in acute care settings have steadily declined in the United States, students who go on to practice in community and public health settings have increased risk of exposure. Following best practices in demonstrating hepatitis B immunity, which include a single challenge dose followed by titer 4 weeks later, would decrease per student costs, wait time, and administrative burden associated with documentation and student counseling.

## 1 INTRODUCTION

Direct patient care clinical experience is an integral part of health sciences educational training. These interactions put both student and patient at increased risk for communicable diseases such as hepatitis B, one of the most common infectious liver diseases. Hepatitis B is a vaccine‐preventable illness caused by the hepatitis B virus (HBV) and spread via blood and blood‐contaminated body fluids. Acute HBV infection in adults is usually self‐limited and will resolve within 6 months, during which time the infection can be transmitted to others. Approximately 5% of infected adults will go on to develop chronic hepatitis B, a lifelong illness that can lead to cirrhosis, liver failure, liver cancer, and premature death (Centers for Disease Control and Prevention [CDC], 2020). In contrast, up to 90% of infants and 50% of children younger than 5 years of age will develop chronic hepatitis B after acute infection (CDC, 2020). To mitigate HBV perinatal transmission and subsequent development of chronic hepatitis B in the United States, in 1991 the Advisory Committee on Immunization Practices (ACIP) recommended routine vaccination at birth, with the first dose to be administered prior to hospital discharge. By 2018, 90.5% of children ages 19–35 months had completed the three‐dose series (Hill et al., 2017). Highly effective and safe, a complete vaccination series confers long‐lasting immunity in over 95% of recipients, precluding the need for a booster dose or treatment in the event of an exposure (World Health Organization, 2020).

Prior to initiating clinical rotations, health sciences students must provide evidence of hepatitis B immunity, such as documentation of a complete vaccination series and/or verification of immunity before clinical placement. Immunity is typically demonstrated by an anti‐hepatitis B surface (anti‐HBs) antibody quantitative serum titer ≥10 mIU/ml. While titers can wane over time, immunologic memory persists for decades, resulting in a rapid and protective anamnestic response to one “challenge” (also referred to as a “booster”) dose of hepatitis B vaccine or infectious exposure. Thus, a titer <10 mIU/ml does not necessarily indicate lack of immunity, especially if obtained more than 8 weeks after the last dose of hepatitis B vaccine was administered (CDC, 2020). This is particularly relevant for newly matriculating health sciences students as the vast majority completed their series in infancy, resulting in an increased likelihood of having low or equivocal titers. Finally, approximately 5% recipients never produce measurable anti‐HBs antibodies, even after two complete rounds of vaccinations. These “non‐responders” remain susceptible to hepatitis B infection and will require additional advisement on necessary preventative precautions, including the use of personal protective equipment and the need for hepatitis B immune globulin (HBIG) for short‐term, passive protection in the event of an exposure.

### 1.1 Follow‐up procedures for low or equivocal titers

ACIP suggests that healthcare personnel working in low‐risk settings and with written documentation of a complete, properly spaced hepatitis B vaccination series do not need routine pre‐exposure antibody testing (Schillie et al., 2018) Those in higher‐risk settings should have pre‐exposure screening; for those with written hepatitis B vaccination documentation and a titer <10 mIU/ml, a challenge dose of hepatitis B vaccine should be administered to elicit an anamnestic response (CDC, 2020). Those with a re‐titer ≥10 mIU/ml 4–8 weeks later should be considered immune and no subsequent testing would be required.

However, approaches to managing students with low/equivocal titers vary by training program. Many programs require students with low/equivocal titers complete another full, 2–3 dose vaccination series, followed by repeat titer 4–8 weeks after the final dose. This process can take between 2 and 8 months, depending on the type of vaccine administered. For example, Heplisav‐B^®^ is a two‐dose schedule with 4 weeks between doses, followed by a titer 4–8 weeks after the second dose (Immunization Action Coalition [IAC], 2020). Engerix‐B^®^ and Recombivax HB^®^ follow a three‐dose schedule, with doses administered at 0, 1, and 6 months, followed by a titer 4–8 weeks after the last dose (IAC, 2020). Determining which students are immune, managing the low/equivocal students through the process of additional vaccinations and titers, and protecting/counseling the non‐responders can be a time‐consuming and costly process for both program administrators and students alike. The purpose of this study was to (1) describe hepatitis B immunity patterns among nursing students entering clinical rotations, (2) discuss student/administrative burden associated with the process, and (3) suggest evidence‐based strategies to streamline the process and reduce costs.

## 2 METHODS

This retrospective, descriptive study was conducted at a large, university‐based college of nursing in the southeast United States that requires students with low/equivocal titers to obtain a full hepatitis B revaccination series and re‐titer. Study activities began after university institutional review board approval. Participants included sophomore nursing students preparing for entry into clinical rotations in summer and fall 2018 and summer 2019.

### 2.1 Data collection and analysis

Per program protocol, students at this institution were required to upload quantitative anti‐HBs titers to CastleBranch, a student‐funded, internet‐based service utilized by higher education institutions to document background checks, immunization records, and drug screens. Students with a low or equivocal anti‐HBs antibody titer were required to (1) document the receipt of dose 1 prior to beginning their first clinical rotation; and (2) document a 2‐3‐dose hepatitis B vaccination series, followed by a re‐titer 4–8 weeks. Data were de‐identified and aggregated by a program administrator prior to analysis; descriptive statistics were generated using Excel.

## 3 RESULTS

A total of 300 students completed the process of hepatitis B immunity documentation in CastleBranch across three semesters (summer and fall 2018; summer 2019) (Table 1).

**TABLE 1:** Demographic characteristics (N = 300)

**Mean age** (range)	21 (19‐48)
	n (%)
**Sex**	
Female	285 (95)
Male	15 (5)
**Race/Ethnicity**	
White	268 (89.3)
Black	13 (4.3)
Asian	5 (1.6)
Hispanic/Latino	1 (0.3)
Two or more races	11 (3.7)
Not reported	2 (0.7)

Of the students who provided documentation of a baseline anti‐HBs quantitative serum titer, only a third reported an anti‐HBs quantitative serum titer ≥10 mIU/ml (Table 2). The remainder reported an anti‐HBs quantitative serum titer <10 mIU/ml (except for three students who did not upload a baseline titer). After documenting their first dose prior to reporting to clinical, these 201 students went on to complete a 2–3 dose hepatitis B vaccination series. At the follow‐up re‐titer 4–8 weeks after the final dose, all but 7 (3.5%) non‐responders demonstrated an anti‐HBs quantitative serum titer ≥10 mIU/ml.

**TABLE 2:** Hepatitis B immunity (N = 300)

**Status**	Summer 2018 (n = 96)	Fall 2018 (n = 93)	Summer 2019 (n = 111)	Total
Baseline anti‐HBs quantitative serum titer ≥10 mIU/ml	29 (30.2%)	32 (34.4%)	38 (34.2%)	99 (33.0%)
Baseline anti‐HBs quantitative serum titer <10 mIU/ml, revaccinated, repeat titer ≥10 mIU/ml	64 (66.7%)	59 (63.4%)	68 (61.3%)	191 (63.7%)
Baseline anti‐HBs quantitative serum titer <10 mIU/ml, revaccinated, repeat titer <10 mIU/ml (non‐responder)	2 (2.1%)	0	5 (4.5%)	7 (2.3%)
No baseline titer, vaccination series completed, follow‐up titer ≥10 mIU/ml	1 (1.0%)	2 (2.2%)	0	3 (1%)

### 3.1 Estimated costs

While this dataset did not include the type of vaccine students with low/equivocal titers received, we used private sector cost estimates for adult hepatitis B vaccination series by brand and number of doses. The CDC estimates a two‐dose series of Heplisav‐B^®^ costs $242.50; a three‐dose series of Engerix‐B^®^ and Recombivax HB^®^ are $185.58 and $183.66, respectively (CDC, 2021). Cost of a quantitative anti‐HBs antibody titer has a wide range, between approximately $8 and $400, and is variably covered by insurance. Cost of a titer at this university student health services was approximately $46, with a $30 lab draw fee. Therefore, a conservative estimate of monetary cost per student after a low/equivocal titer would be $259.66 with immunity demonstrated approximately 7–8 months later; highest costs would be $318.50 with immunity demonstrated in 4–8 weeks. Therefore a single challenge dose, as compared to a complete 2 or 3 dose series, represents a minimum cost savings of $125 per student ($21,875 cumulative). These costs do not include the expense of a clinic visit (if required) and associated vaccine administration costs (e.g., supplies, vaccine storage, staff training). Non‐monetary costs included the burden students faced in negotiating a complex policy during the first semester of their clinical rotations and significant time spent on health care appointments. Administrative costs included time dedicated to student follow‐up (e.g., tracking of student documentation, email prompts/reminders), database updates, and counseling for non‐responders.

## 4 DISCUSSION

Health sciences educational training requires direct patient contact, increasing the risk for HBV exposure (CDC, 2020) and requiring that evidence‐based policies must be in place to ensure a safe environment for both students and patients. While rates of exposure to hepatitis B in acute care settings have steadily declined in the United States, students who go on to practice in community and public health settings have increased risk of exposure, as they serve communities with higher rates of hepatitis B and lower rates of vaccination (e.g., immigrant [Kue & Thorburn, 2013], homeless [McCosker et al., 2022], and substance abuse [Kulikowski & Linder, 2018]), underscoring the importance of determining a student's status early in their career. Current evidence indicates that even among individuals who completed a hepatitis B vaccination series 30 years prior and with initial anti‐HBs levels below 10 mIU/ml, 90% will demonstrate protective levels ≥10 mIU/ml 4 weeks after a single challenge dose of hepatitis B vaccine (Bruce et al., 2016). By following current ACIP guidelines, approximately 175 students in this study would have been able to demonstrate immunity in a minimum of four weeks, at significantly decreased cost, after a single challenge dose and repeat titer. These results will inform research focused on a prospective evaluation of efficacy and cost savings of a single challenge dose/re‐titer in demonstrating hepatitis B immunity for nursing students.

## CONFLICT OF INTEREST

The authors have no conflicts of interest to disclose.

## Data Availability

The data that support the findings of this study are available from the corresponding author upon reasonable request.
